# Investigating Excited States and Absorption Spectra of the Poly-cyclopenta-dithiophene-benzothiadiazole Oligomers (Poly-CPDTBT)—A Theoretical Study

**DOI:** 10.3390/molecules29225348

**Published:** 2024-11-14

**Authors:** Jun Wang, Yuting Huang, Yajing Wang, Bo Durbeej, Lluís Blancafort

**Affiliations:** 1 School of Chemistry and Chemical Engineering, Huaiyin Normal University, No. 111 West Changjiang Road, Huaian 223300, China; 2 Division of Theoretical Chemistry, IFM, Linköping University, SE-581 83 Linköping, Sweden; 3 The Institute of Computational Chemistry and Catalysis and Department of Chemistry, University of Girona, C/M.A. Capmany 69, 17003 Girona, Spain; lluis.blancafort@udg.edu

**Keywords:** Poly-CPDTBT, transition density matrix, absorption spectrum, local and charge transfer excitation

## Abstract

Poly-CPDTBT, as typical low-band gap copolymers, have potential applications in organic bulk heterojunction solar cells. To have a clear picture of its excited-state processes, the first task is to understand their excited states, in particular, electronic character and relevant optical absorption. Herein, the low-lying singlet excited states of Poly-CPDTBT oligomers were investigated via Algebraic Diagrammatic Construction Second Order (ADC(2)) and time-dependent density functional theory (TDDFT) method with several functionals. Six CPDTBT_N_ (N = 1–6) oligomers were taken as prototypes to study their excited states in detail. The results provide interesting clues to extrapolate the photophysical properties of such polymers with potential applications in photovoltaic materials. The result provided by ωB97XD functional gives good agreement with the experiment result. The vertical excitation energies of the four lowest excited states decrease almost linearly with increasing polymerization degree (N) for CPDTBT_N_ (N = 1–6). The transition density analysis indicates that the local excitations (LE) and the short-distance charge transfer (CT) excitations between two adjacent CPDT and BT units are dominant for low-lying excited states for short oligomers. For the long-chain oligomers (trimer to hexamer), the transition density shows a ladder (or zigzag) pattern along the diagonal blocks at the planar geometry. For long oligomers, the whole chain is involved in the transitions, and the CT excitations only exist between two adjacent CPDT and BT units. The present work provides a valuable basis for understanding the excited-state processes of Poly-CPDTBT and other conjugated polymers that conduct solar energy conversions, which has great significance for the development of new solar cells.

## 1. Introduction

Conjugated polymers with excellent optoelectronic and mechanical properties have attracted great research interest due to their potential applications in many areas, such as photovoltaic materials and optical switches [1,2,3,4,5,6]. Especially in recent years, the design of novel π-conjugated polymers to obtain highly efficient low-cost organic solar cells has received broad interest in both academic and commercial communities [7,8]. A common idea to improve the photovoltaic performance of conjugated polymers is the construction of so-called “D-A copolymers”, which include the electron donor (D) and electron acceptor (A) units in the same polymer chain.

Poly-cyclopenta-dithiophene-benzothiadiazole is among such low-band gap copolymers with potential applications in organic bulk heterojunction solar cells [9,10,11,12]. Poly-CPDTBT consists of two units: an electron-rich (cyclopentadithiophene, CPDT) and an electron-poor (benzothiadiazole, BT) unit. The Poly-CPDTBT-family materials, introduced by Brabec [9] and co-workers in 2006, received great attention due to their very good performance. Great efforts were made to further improve the light-to-current conversion efficiency of organic solar cells based on Poly-CPDTBT-series materials [13,14,15,16,17,18,19,20]. For instance, many studies focused on the modification of electronic properties of polymers by introducing new functional groups or controlling device morphology by solvent additives [14,19,21,22,23].

As it is very difficult to obtain a breakthrough when only the traditional protocols mentioned above are employed, understanding the photophysical and photochemical processes is extremely important for further improvements of the photovoltaic feature of the Poly-CPDTBT-family materials. Generally speaking, the formation and migration of excitons in conjugated polymer is an essential research topic [7,24]. At the same time, it is very challenging to understand these processes due to system complexity [25,26,27,28,29,30]. 

To obtain a clear picture of the excited-state processes of Poly-CPDTBT and other conjugated polymers, the first task is to understand their excited states, particularly their electronic character and relevant optical absorption. In the past decades, many theoretical efforts have been made to investigate the excited states of conjugated polymers [12,31,32,33]. These studies focused on different topics. For example, the character of the excited states and the absorption spectrum for low-band-gap conjugated polymer have been systematically investigated by several electronic-structure calculations (from semiempirical to density functional theory) [12,18,31,32,34]. The photoinduced charge-transfer dynamics between conjugated polymers and electron–acceptor compounds also invoked wide discussions [18,26,27,35,36,37].

However, fundamental difficulties still exist in the theoretical analysis of excited states of conjugated polymers. First, accurate electronic-structure calculations with reasonable computational cost are very challenging for molecular excited states of complex systems. Polymer systems and even their oligomers, composed of a huge number of atoms, are beyond the treatable limit of high-level correlated methods. Second, although TDDFT is very popular in dealing with large systems, taking advantage of its accurate description of valence excitations, it suffers severely from the improper description of charge-transfer (CT) states [38] and the lack of double excitations [38,39]. Third, the analysis of the excited state character of such copolymers is not a trivial task because different transition components, namely, local excitations (LE) and charge transfer (CT) excitations within D and A units, are involved. The characterization of excited states should take all possible excitations (LE and CT within all interacting units) into account. In the past years, several groups [24,40,41,42,43,44,45,46,47,48] developed various approaches essentially based on transition density matrix to analyze the LE and CT contributions based on an interaction picture of predefined units of complex systems.

The purpose of this work is to address three important issues. First, we try to examine the performance of different electronic structure methods in the treatment of CPDTBT oligomer systems. This provides useful information for the choice of suitable theoretical methods to deal with similar large conjugated copolymers. Second, we wish to clarify the important contributions of electronic transitions for each excited state. This helps us to set up a detailed analysis of the electronic character by using the transition density matrix. Third, we wish to investigate the dependence of excited-state properties on the degree of polymerization. This guides us in deriving the excited-state electronic structure by using the extrapolation of polymerization approaches to infinity. To achieve this goal, oligomers CPDTBT_N_ with different numbers of units (N = 1–6) were investigated. To examine the performance of different computational methods, benchmark calculations were carried out for the short oligomers (N = 1–2) using ADC(2) [49,50] and TDDFT [38,51,52,53] with several functionals, i.e., B3LYP [54,55] (20% HF exchange), PBE0 [56,57] (25% HF exchange), BHandHLYP [55,58,59] (50% HF exchange), CAM-B3LYP [60] (range-separated functional), and ωB97XD [61] (functional with long-range correction and empirical dispersion). For the long oligomers (N = 3–6), only TDDFT was employed. The absorption spectra were calculated for the above oligomers. The electronic character of the low-lying singlet excited states was examined by a method based on the transition density analysis. The extrapolation to N infinity provides useful clues to understanding the excited-state properties of the polymers. This work provides useful information for understanding the formation of excitons in conjugated polymers. 

## 2. Model Construction

**Scheme 1:**
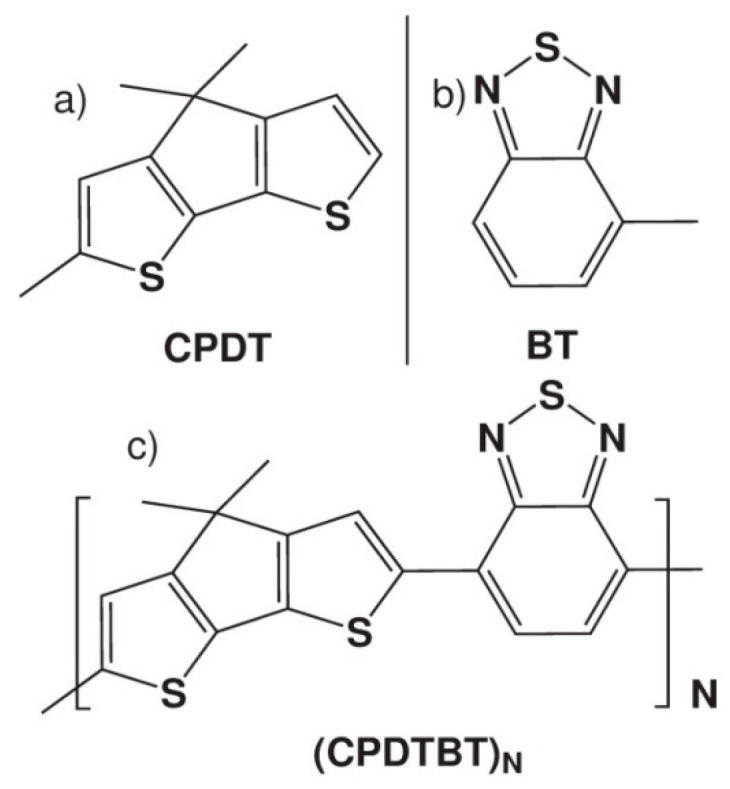
Structures of (**a**) CPDT, (**b**) BT, and (**c**) copolymers (CPDTBT)_N_.

Accurate electronic-structure calculations of large polymer systems with a large number of atoms are, in principle, not feasible yet. To explore the relevant properties of polymers, many studies [28,29,62] selected corresponding oligomers as the prototypes. Within this approach, the key features of polymers may be obtained by extrapolation from those of short oligomers. In the current study, we took this research strategy and started the investigation from the short oligomers of Poly-CPDTBT copolymers. A series of Poly-CPDTBT oligomers with different lengths (CPDTBT_N_, N = 1–6) were considered, i.e., monomer, dimer, trimer, tetramer, pentamer, and hexamer. Every repeated moiety of CPDTBTN consists of two units: an electron-rich cyclopentadithiophene (CPDT) donor and an electron-poor benzothiadiazole (BT) acceptor (Scheme 1).

**Scheme 2:**
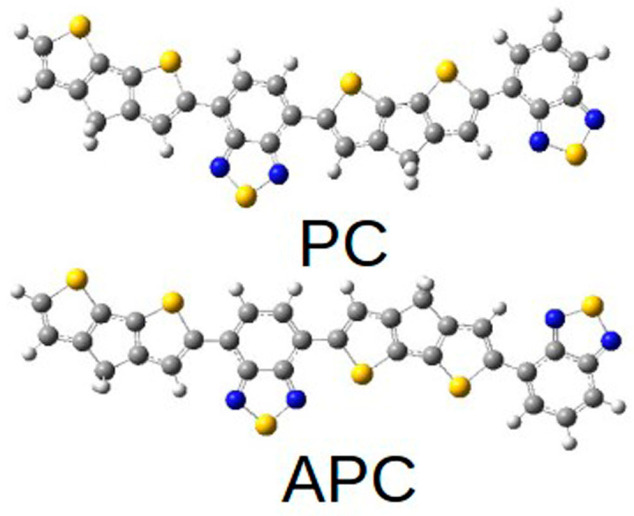
Two configurations of dimer: parallel configuration (PC) and anti-parallel configuration (APC).

In addition, the orientation of two adjacent repeated moieties results in two configurations: parallel configuration (PC) and anti-parallel configuration (APC). For example, the dimer has two different configurations: PC and APC (Scheme 2). For all CPDTBT_N_ (N = 2–6), we consider two isomers where the relative configurations of adjacent moieties are all PC or APC. 

## 3. Results

### 3.1. Equilibrium Geometry of Ground State

**Figure 1:**
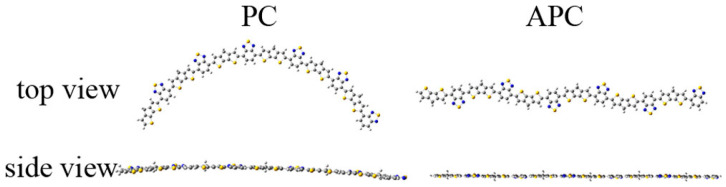
Top and side views of hexamer with PC and APC.

The ground-state minimum geometry of the monomer was optimized under the C_s_ point group symmetry constraint at the BHandHLYP/def2-SVP level of theory. Based on this planar geometry, we recalculated the geometry optimization at the BHandHLYP/def2-SVP level of theory without any symmetry constraint. The geometry obtained with C_1_ symmetry is almost identical to that of C_s_ symmetry. The optimization at the B3LYP/def2-SVP level gives almost the same ground-state geometry, implying a weak dependence of the ground-state geometry on the functional selection. For CPDTBT_N_ (N = 2–6), i.e., dimer, trimer, tetramer, pentamer, and hexamer, the ground-state minimum geometries for two configurations (PC and APC) were all optimized at the BHandHLYP level. For the dimer and trimer, the def2-SVP basis set was used. For tetramer to hexamer, the SV basis set was used. Results indicate that the ground-state minimum geometries of CPDTBT_N_ (N = 1–6) are all planar structures for both configurations (Figure 1). For long oligomers, such as hexamer, two configurations (PC and APC) are different from the top view. PC displays an arc structure, while APC displays a linear structure (Figure 1). As expected, the APC isomer is more stable, while the energy difference between the two configurations is not dramatic (~5 kcal). Because PC and APC isomers share many similarities in their excited-state properties, such as vertical energies, oscillator strengths, and electronic characters, we mainly focus on APC isomers in the following discussion. All details on PC isomers can be found in the Supplementary Materials.

### 3.2. Excited-State Energies and Character

#### 3.2.1. Benchmark Calculations and Short Oligomers

Monomer and dimer were taken as prototype systems to check the performance of different methods. The electronic character of low-lying excited states and the transition density analysis were considered in these benchmark calculations.

At the equilibrium geometry of the monomer, the excited states were calculated at different levels of theory, i.e., TDDFT with different functionals (B3LYP, PBE0, BHandHLYP, ωB97XD, and CAM-B3LYP) and ADC(2). In the TDDFT calculations (Table 1), the vertical excitation energies of the lowest excited states increase with the inclusion of more Hartree-Fock (HF) exchange. For instance, the first excited singlet state S_1_ energies are 2.51 eV (B3LYP, 20% HF exchange), 2.64 eV (PBE0, 25% HF exchange), and 3.04 eV (BHandHLYP, 50% HF exchange). The range-separated functional CAM-B3LYP and ωB97XD (including dispersion correction) with 6-31G* basis set give similar results (3.07 eV and 3.09 eV, respectively) as BHandHLYP/def2-SVP.

The vertical excitation energy (3.33 eV) of S_1_ obtained at the ADC(2) level is higher than the TDDFT results. Overall, for the two low-lying singlet states (S_1_ and S_2_), TDDFT calculations with BHandHLYP, CAM-B3LYP, and ωB97XD functionals seem to provide similar results with the ones at the ADC(2) level. In addition, the S_1_-S_4_ states are dominated by single excitations, and the contribution of double excitations is below 12% at the ADC(2) level (see Table S1, Supplementary Materials (SM)).

The oscillator strengths (OSs) and the transition contributions of the S_1_ and S_2_ states are listed in Table 1. The electronic character of the two lowest excited states (S_1_ and S_2_) is independent of the choice of the methods/functionals, while inconsistent results are obtained for the higher states above S_3_. In the current work, we mainly focus on the two lowest excited states (S_1_ and S_2_) since a reasonable description of high-lying excited states is generally not possible. In addition, the lowest two states (S_1_ and S_2_) are responsible for the absorption of the monomer due to their high OSs. 

**Figure 2:**
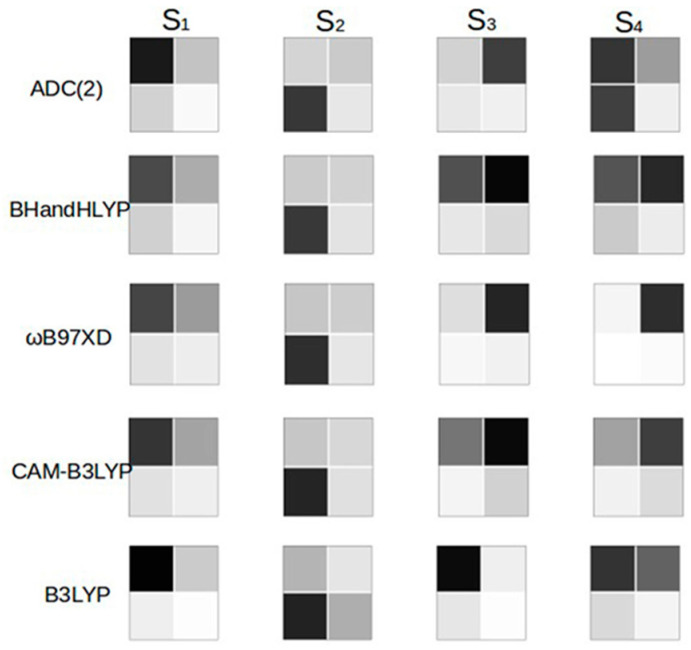
The transition density analysis of S_1_–S_4_ for monomer with APC at the ADC(2)/def2-SVP, BHandHLYP/def2-SVP, ωB97XD/6-31G*,CAM-B3LYP/6-31G*, and B3LYP/def2-SVP levels.

The electronic character of the S_1_ and S_2_ states was further considered by examining the involved orbitals (Table 2) and the transition density analysis at the ADC(2) and TDDFT levels (Figure 2). In the transition density plots, the squares on the diagonal starting from the bottom left represent LE contributions, and the remaining squares represent CT states. The darker grey color represents a larger contribution to the excited state wave function. Further details of the transition density analysis used in this paper are described in the section “Computational Methods”. At the equilibrium geometry, three frontier orbitals (HOMO-1, HOMO, LUMO+1) are extended over the whole molecule. In contrast, the density of the LUMO is located mainly at the BT unit, although some delocalization is observed. For the transition density analysis, ADC(2) gives almost the same result as TDDFT with the B3LYP, BHandHLYP, CAM-B3LYP, and ωB97XD functionals. To describe the excitation, we use a nomenclature where D(X) and A (X) indicate that X is denoted as the donor and acceptor, respectively. The main contribution of the S_1_ excitation in the monomer is D(CPDT) → A(BT) CT excitation (~50%), while the other two LE [D(CPDT) → D(CPDT): 16% and A(BT) → A(BT): 27%] transitions also contribute to the wave function. The second excited state, S_2_, is mainly composed of both BT → BT LE excitations and CPDT → CPDT LE excitations (Figure 2). 

The dimer was taken as the second prototype system to check the performance of different methods. Four frontier orbitals (HOMO-1, HOMO, LUMO, and LUMO+1) of the dimer with APC conformation are displayed in Table 2. Two orbitals (HOMO-1 and HOMO) are delocalized over the whole molecule, while both LUMO and LUMO+1 show large densities on the BT unit (Table 2). For the low-lying states (S_1_ and S_2_), the calculations at TDDFT (BHandHLYP, CAM-B3LYP, and ωB97XD) and ADC(2) levels also have many similarities, including their oscillator strengths, electronic character, transition contributions, and involved orbitals (Table 3).

**Figure 3:**
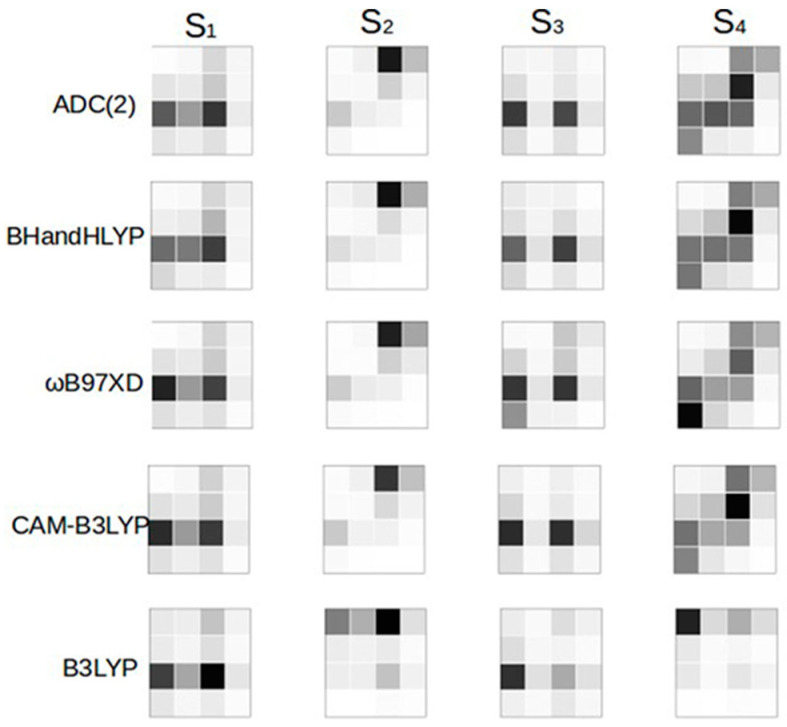
The transition density analysis of S_1_-S_4_ for dimer with APC at the ADC(2)/def2-SVP, BHandHLYP/def2-SVP, ωB97XD/6-31G*, CAM-B3LYP/6-31G*, and B3LYP/def2-SVP levels.

**Figure 4:**
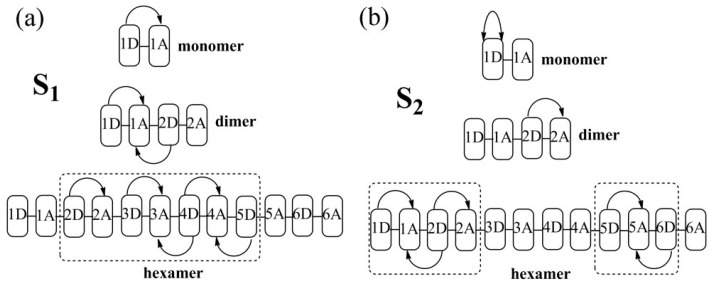
The main contributions of excitations for the two lowest excited states (**a**) S_1_ and (**b**) S_2_ in the monomer, dimer, and hexamer with APC.

At all levels, S_1_ is the bright state with a large OS. It is responsible for the strong photo-absorption of the Poly-CPDTBT dimer. Similar to the monomer, the vertical excited energy of S_1_ (2.61 eV) obtained at the ADC(2) level is higher than the TDDFT data. The vertical excitation energy of S_1_ gives the following order: ωB97XD (2.49 eV) > CAM-B3LYP (2.40 eV) > BHandHLYP (2.35 eV). The S_1_ wave function is described by a mixture of LE excitation and CT excitation (Figure 3). Two CT excitations (1D → 1A and 2D → 1A) are predominant, while two LE (1A → 1A and 2D→2D) excitations at two central units should also be taken into account (Figure 4). In contrast, two LE excitations (1D → 1D and 2A → 2A) seem not very important, and the 2D → 2A excitation is much weaker than another CT excitation (Figure 4). The S_2_ state displays a strong mixture of the LE and CT excitations. Notice that the low-lying CT excitations take place between two adjacent CPDT and BT units, but there are no relevant long-distance CT excitations (Figure 3). This results in a ladder (or zigzag) pattern along the diagonal blocks in the transition density analysis blocks (Figure 3). For the low-lying states of the dimer, the contributions of double excitation configurations are still quite low at the ADC(2) level (<15%).

Based on the preceding discussion, the CT excitations between two adjacent units in the dimer are important for the low-lying states (S_1_, S_2_). Such CT excitations determined by two adjacent units may be viewed as the “fixed-distance” electronic transition. For longer CPDTBT oligomers, fixed-distance CT excitations should be essential. The longer distance CT excitations between non-adjacent units can be properly ignored for low-lying excited states because their excitation energy is too high. That is to say that the “CT excitation distance” is independent of the chain length. From the comparison of the results obtained by TDDFT (BHandHLYP, CAM-B3LYP, and ωB97XD) and ADC(2), we may conclude that TDDFT (BHandHLYP, CAM-B3LYP, and ωB97XD) gives a reasonable description of the fixed-distance CT excitations. Therefore, it should be safe to use TDDFT (BHandHLYP, CAM-B3LYP, and ωB97XD) to investigate the excited states of the longer oligomers.

#### 3.2.2. The Oligomer CPDTBT_N_ (N = 3–6)

The vertical excitation energies and oscillator strengths obtained with the BHandHLYP, CAM-B3LYP, and ωB97XD functionals for the four lowest states (S_1_ and S_4_) of CPDTBT_N_ (N = 3–5) in APC conformation are listed in Table 4. Ground-state geometries were optimized at the BHandHLYP/def2-SVP level. The results for the hexamer (N = 6) are listed in Table 5. For the excitation energies and OSs, the three functionals give similar results for trimer to hexamer. S_1_ is the bright state with the largest OS. In addition, similar to the monomer and dimer, the vertical excited energy of S_1_ for all species also follows the order ωB97XD (1.89 eV) > CAM-B3LYP (1.77 eV) > BHandHLYP (1.67 eV). 

The orbitals of CPDTBT_N_ (N = 3–6) have common features; thus, we only discuss the hexamer (N = 6) here and put the others in the SM (Tables S2–S4). The four frontier orbitals (HOMO-1 to LUMO+1) for the hexamer are displayed in Table S5. Similar to the short oligomers (but not the monomer), the HOMO-1 and HOMO are delocalized over the whole system, while the densities of the LUMO and LUMO+1 are mainly located at the BT units. From the view of the whole chain, HOMO and LUMO are distributed over the whole chain, while a large density is observed in the central part instead of two ends. However, the densities of HOMO-1 and LUMO+1 at the center of the chain are very low.

**Figure 5:**
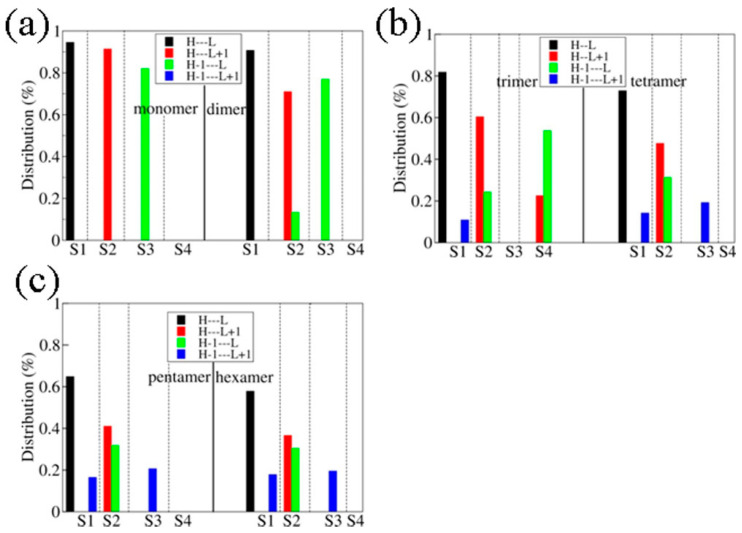
Distribution (%) of the four transitions (HOMO → LUMO, HOMO → LUMO+1, HOMO-1 → LUMO, and HOMO-1 → LUMO+1) on the excited states (S_1_, S_2_, S_3_, and S_4_) for the six oligomers of APC: (**a**) monomer and dimer; (**b**) trimer and tetramer; (**c**) pentamer and hexamer. H, L, H-1, and L+1 denote HOMO, LUMO, HOMO-1, and LUMO+1, respectively.

At the planar geometry, the S_1_ is mainly dominated by the HOMO → LUMO transition for monomer and dimer, while the contribution of this component decreases monotonically when increasing the polymerization degree (Figure 5). At the same time, the contribution of the HOMO-1 → LUMO+1 transition starts to become important for the S_1_ state of long oligomers CPDTBT_N_ (N = 3–6). Character change was also found for the S_2_ state (Figure 5). For the monomer, the major component is the HOMO → LUMO+1 transition (91.4%), while its contribution decreases gradually from monomer to hexamer (41%). Meanwhile, the HOMO-1 → LUMO transition starts to appear from the dimer on, increasing gradually with increasing polymerization degree. For the pentamer and the hexamer (Figure 5), the HOMO → LUMO+1 and HOMO-1 → LUMO transitions give almost similar contributions to the S_2_ state. 

In addition, CPDTBT_N_ (N = 2–6) in the PC configuration were also considered. Results of the energies, oscillator strengths (OS) and contributions of four lowest excited states for these oligomers can be seen in Tables S6–S10.

**Figure 6:**
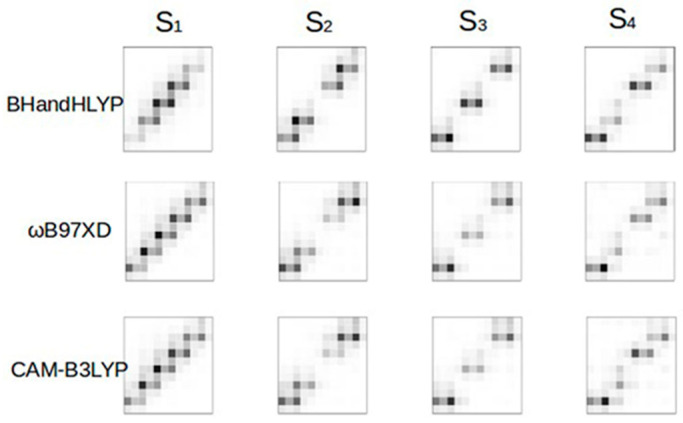
Transition density analysis of S_1_-S_4_ for hexamer with APC at the BHandHLYP/def2-SVP, ωB97XD/6-31G*, and CAM-B3LYP/6-31G* levels.

The transition density analysis of the excited states for the Poly-CPDTBT hexamer was performed (Figure 6). Similar to the dimer, the transition density of the S_1_ state shows a ladder pattern along the diagonal blocks for the hexamer. The S_1_ state shows CPDT → BT CT transitions, which exist only between two adjacent CPDT and BT units. There are almost five main contributions (2D → 1A, 2D → 2A, 3D → 2A, 3D → 3A, 4D → 3A, 4D → 4A, and 5D → 4A) in the excitations of S_1_. (Figure 4) The whole chain is involved in the transitions, while the terminal units show less relevance (Figure 6). Contrary to the S_1_ state, the transition to the S_2_ state displays a node in the middle of the chain. Additionally, transition densities of dimer and hexamer in the PC configurations were also considered, which can be seen in Figures S1 and S2. 

#### 3.2.3. Simulated Absorption Spectra 

**Figure 7:**
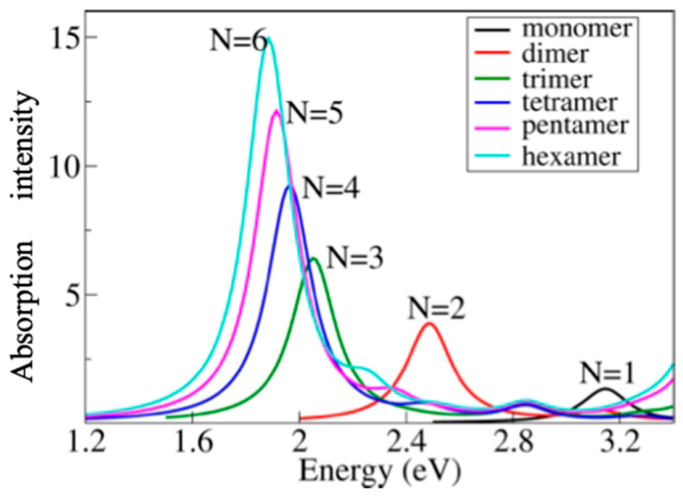
Simulated absorption spectra of CPDTBT_N_ (N = 1–6) in APC conformation obtained at the ωB97XD/6-31G* level.

The simulated absorption spectra for the oligomer CPDTBT_N_ (N = 1–6, APC) are given in Figure 7. From monomer to hexamer, the lowest S_1_ state is always responsible for the absorption spectra. The contributions of other excited states are rather minor. Similar behavior was also found in other linear conjugated systems that are treated as J aggregates [63]. In addition, an enhancement of the absorption intensity and a red shift of the peak position are observed with the larger polymerization degree. The absorption position of the monomer (3.1 eV) is far from that of the dimer (2.5 eV), while such a difference becomes lower between long oligomers.

#### 3.2.4. Extrapolation of the Vertical Excited Energy of S_1_

Key features of polymers may be obtained by extrapolation from those of short oligomers. Several approaches [28,32,64,65,66] were proposed to extrapolate the excitation energy of long polymers. Following the particle-in-a-box model, the excitation energy is assumed to be linearly dependent on 1/N [28,67],

(1) $$E_{i}=A_{i}+\frac{B_{i}}{N}$$

Alternatively, the S_1_ excitation energy can be obtained by Kuhn’s equation [65]:

(2) $$E_{i}=A_{i}\sqrt{1+B_{i}\cos\frac{\pi }{N+1}}$$

This equation treats the polymer as an ensemble of several interacting chromophores. The derivation of this equation is found in reference [68]. In this work, both equations were employed in the extrapolation and the fitting parameters and extrapolated energy of S_1_ (E_1_) using the above two equations at the BHandHLYP/def2-SVP, CAM-B3LYP/6-31G*, and ωB97XD/6-31G* levels and are listed in Table 6. The extrapolation to N → ∞ should, in principle, yield the S_1_ excitation energies of the polymers. For these three functionals, the extrapolated excitation energies of S_1_ based on the particle-in-a-box model (Equation (1) are lower than those obtained with Kuhn’s equation (Equation (2)). In general, the fitted models provide extrapolated excitation energies that are lower than the experimental value of 1.71 eV [12,31]. The best agreement with the experiment is obtained by applying Kuhn’s equation to the ωB97XD/6-31G* functional (S_1_: 1.67 eV).

## 4. Discussion

The pure functional underestimates the energy of CT states, and the percentage of HF exchange in the hybrid functionals has a great influence on the energy of CT excitation [38]. Normally, a hybrid functional with a suitable amount of HF exchange or a range-separated functional seems to provide a better result. Thus, it is necessary to try different functionals until satisfactory data are obtained. For monomer and dimer, the BHandHLYP, CAM-B3LYP, and ωB97XD methods give a similar result to the ADC(2) method for the two lowest excited states (S_1_ and S_2_). The electronic character analysis of monomer and dimer indicates that the CT contributions are dominated by transitions between adjacent CPDT and BT units. Based on this observation, we expected that only adjacent CPDT → BT and BT → CPDT CT excitations are important for the low-lying states of the longer Poly-CPDTBT oligomers. Since a consistent description of such short-distance CT excitation is obtained with BHandHLYP, CAM-B3LYP, ωB97XD, and ADC(2), we propose that the BHandHLYP, CAM-B3LYP, and ωB97XD functionals are suitable choices for predicting the excited states of the CPDTBT_N_ (N = 3–6) oligomers.

For highly conjugated systems, double excitation may play an important role, as pointed out by Dreuw and co-workers [39]. Since ADC(2) describes the double-dominated excitations at 0th order in terms of the fluctuation operator, the results of double excitations obtained using ADC(2) are possibly not reliable. For the low-lying states of current systems, the contribution of double excitation at the ADC(2) level is less than ~15% for monomer and dimer, ruling out their possible involvement. For long oligomers, it is difficult to estimate the contribution of double excitation due to the size of the system. This represents a very important topic for future research. However, in the current stage, it is possible to have a qualitative understanding of the excitonic states of oligomers, at least for the excited states that are dominated by single excitations. 

At first glance, the contribution of the lowest excited state S_1_ of oligomers seems to be well described by a mixture of intra-unit LE and inter-unit CT excitations. This raises the question of whether it is possible to parametrize a diabatic model Hamiltonian based on the diabatic LE and CT excitations and the electronic couplings among them. This may facilitate the modeling of larger oligomers, and it would be a first step toward modeling exciton dynamics with an effective Hamiltonian. However, the construction of such a diabatic Hamiltonian from electronic-structure calculations has turned out to be very challenging in this case. Attempts to use the diabatization method developed by Subotnik [69,70,71] (in the Q-Chem package [72]) have been unsuccessful because the resulting diabatic states were not properly localized. This is due to the presence of other states that, in the diabatic picture, correspond to higher LE or CT excitations. They act as intruder states and make it impossible to obtain a complete diabatic basis for the states under consideration. Attempts to set up an approximate diabatic model based on calculations on individual units and estimation of the couplings in the oligomer [73], or the renormalized exciton model [74,75,76], have also been unsuccessful. Thus, the parametrization of a diabatic Hamiltonian remains a challenging task for future work.

## 5. Conclusions

In summary, the two low-lying excited states (S_1_ and S_2_) of Poly-CPDTBT oligomers were investigated using various theoretical methods, ranging from ADC(2) to TDDFT with different functionals (B3LYP, PBE0, BHandHLYP, CAM-B3LYP, and ωB97XD). Six oligomers, including monomer, dimer, trimer, tetramer, pentamer, and hexamer, were considered in this paper. For short oligomers, the TDDFT methods (BHandHLYP, CAM-B3LYP, and ωB97XD) give consistent results with the ADC(2) method. Therefore, BHandHLYP, CAM-B3LYP, and ωB97XD functionals were selected to investigate the excited states of long Poly-CPDTBT oligomers. Particularly, results obtained by ωB97XD give good agreement with the experiment result.

The vertical excitation energies of the four lowest excited states almost linearly decrease with increasing polymerization degree (N) for CPDTBT_N_ (N = 1–6). The extrapolation of N → ∞ for the polymer gives the vertical excitation energies of the low-lying excited states. Our result shows good agreement with experiment values.

The transition density analysis of the excited states was performed. For short oligomers (monomer and dimer), the LE excitations and the short-distance CT excitations between two adjacent CPDT and BT units are dominant for low-lying excited states. Due to the absence of long-distance CT transitions, we expect that only such adjacent CPDT → BT and BT → CPDT CT excitations are important for longer Poly-CPDTBT oligomers. Since the BHandHLYP, CAM-B3LYP, ωB97XD, and ADC(2) methods give a consistent description of such short-distance CT excitation, these three functionals should be a suitable choice for the calculations of excited states for long CPDTBT_N_ (N = 3–6). For the long-chain oligomers (trimer to hexamer), the transition density shows a ladder (or zigzag) pattern along the diagonal blocks at the planar geometry. For long oligomers, the whole chain is involved in the transitions, and the CT excitations only exist between two adjacent CPDT and BT units.

This work provides a solid basis for the further investigation of excited-state processes of conjugated polymers. Particularly, it also points out the dilemma of excited state calculations because the results are strongly dependent on the method selection. Thus, it is extremely important to develop novel electronic-structure theories for the accurate understanding of optical–electronic properties of Poly-CPDTBT and other conjugated systems.

## 6. Computational Methods 

The excited states of the Poly-CPDTBT oligomers with two configurations were investigated by the time-dependent density functional theory (TDDFT) method, including BHandHLYP, CAM-B3LYP, and ωB97XD functionals. The ADC(2) method was taken as a benchamark to examine the performance of TDDFT with different functionals in the treatment of short oligomers. The calculations at the TDDFT/CAM-B3LYP and TDDFT/ωB97XD levels were made with Gaussian 09 software [77], and all other calculations were performed within the TURBOMOLE 6.5 package [78].

The ground-state equilibrium geometries of CPDTBT_N_ (N = 1–6) were optimized at the BHandHLYP level. A large number of geometries were generated from the Wigner distribution function of the normal modes of these optimized geometries. The single-point TDDFT/ωB97XD/6-31G* calculations were performed at each sampled geometry. After the collection of vertical excitation energies and oscillator strengths (OSs), a Gaussian broadening function with half-height full-weight 0.1 eV was used to obtain the absorption spectra.

For CPDTBT_N_ (N = 1–6), the LE and CT transitions within D and A units are involved in the electronically excited states. Here, the LE component refers to the transition within a single CPDT or BT unit, and the CT component is denoted as the electronic transition from one unit to another. It is necessary to analyze the excited-state character and all possible excitations (LE or CT) to gain insight into the excited states. 

The theoretical method based on the single-electron transition density matrix [24,47,79] was employed to analyze the excited-state character. Since this method was discussed in detail previously [29,40,45,46,48,80,81,82], we only outline the main idea here. The one-electron transition density matrix from the *s*^th^ MO to the *r*^th^ MO is given as

(3) $$T_{rs,EG}=<E|{a_{r}}^{+}a_{s}|G>$$

where |E> and |G> refer to the excited and ground state many-electron wave functions, respectively. The a_r_^+^ (a_s_) is a creation (annihilation) operator of an electron at the *r*^th^ (*s*^th^) MO. The transition density matrix T^[MO]^ in the atomic orbital (AO) basis is obtained by

(4) $$T^{\left[\mathrm{AO}\right]}=CT^{\left[\mathrm{MO}\right]}C^{t}$$

where C is the MO coefficient matrix. In principle, the total transition probability between two atoms should be given by simply adding all contributions of relevant AO transitions. Due to the non-orthogonality of the AO basis, $T^{EG,[AO]}$ is transformed with Löwdin orthogonalization [47]:

(5) $$T^{EG,[LO]}={\left(S^{\left[AO\right]}\right)}^{\frac{1}{2}}T^{EG,[AO]}{\left(S^{\left[AO\right]}\right)}^{\frac{1}{2}}$$

where the transition density matrix $T^{EG,[LO]}$ is formed on the orthogonal basis. Thus, the transition probability from atom b to atom a simply becomes

(6) $${B_{ab}}^{EG}=\sum_{i\in a,j\in b}{\left(T^{EG,[LO]}\right)}_{ij}^{2}$$

where i, j are the indices of the atomic basis, and a, b are the indices of atoms. Thus, the B → A transition probability from fragment B to fragment A of a molecule is given by

(7) $${\Omega_{AB}}^{EG}=\sum_{a\in A,b\in B}{B_{ab}}^{EG}$$

The cases of A = B and A≠B in Equation (7) represent the LE (A → A and B → B) excitations and the CT (A → B and B → A) excitation probabilities, respectively. In this way, the contributions of all LE and CT excitations are viewed directly for an excited state.

Similar to previous works [29,80,81], the analysis at the ADC(2) level takes the single-excitation response coefficient as the transition density. This approach works reasonably well when excited states are dominated by singly excited configurations [29,40,80].

**Scheme 3:**
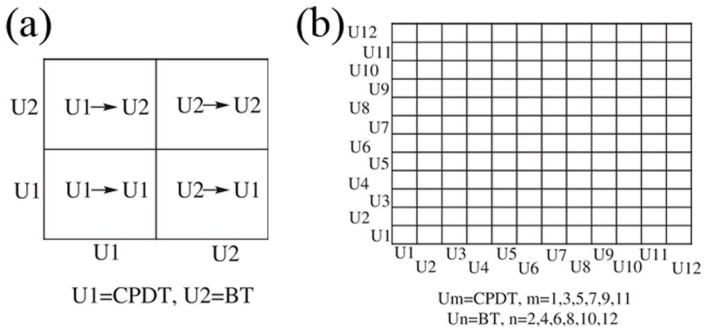
The classification of the electronic transitions for different oligomers. (**a**) Monomer: U1 and U2 denote the CPDT and BT units, respectively. U1 → U1 and U2 → U2 represent the local excitation at U1 and U2, respectively. U1 → U2 and U2 → U1 are the charge transfer excitations between U1 and U2. (**b**) Hexamer: each square shows the transition from the Ux (horizontal) to Uy (vertical) unit.

The rule for partitioning the whole system is explained in Scheme 3. A gray scale is filled into each square, representing the transition probability from the corresponding horizontal block to the vertical block (Scheme 3a,b). A darker gray color indicates a larger transition probability. For example, in the monomer case, U1 and U2 (Scheme 3a) denote BT and CPDT units, respectively. U1 → U1 and U2 → U2 represent the local excitations at U1 (CPDT) and U2 (BT), respectively. U1 → U2 and U2 → U1 define the BT → CPDT and CPDT → BT CT excitations, respectively. For hexamer (Scheme 3b), there are twelve units (6 CPDT and 6 BT). The diagonal squares (from left/down to right/up) indicate the LE excitations (CPDT/BT to CPDT/BT itself), while the other squares indicate the CT excitations that include the ones between two adjacent units and two long-distance separated units. Transition densities of all the contributions for monomer and dimer have been obtained (Tables S11 and S12) The transition density analysis was performed by the program developed by Lan, which was used in our previous work [81]. 

## Figures and Tables

**Table 1:** The vertical excitation (VE) energies (eV), oscillator strengths (OSs), and contributions of four lowest excited states for monomer at the ADC(2)/def2-SVP, BHandHLYP/def2-SVP, ωB97XD/6-31G*, and CAM-B3LYP/6-31G* levels. H and L indicate HOMO and LUMO, respectively.

Method	Excited States	VE	OS	Contribution
ADC(2)	S_1_	3.33	0.63	H → L (0.94)
	S_2_	4.44	0.36	H → L+1 (0.83)
	S_3_	4.62	0.05	H-3 → L (0.37) H-2 → L (0.30)
	S_4_	4.81	0.12	H-4 → L (0.48) H-2 → L (0.17)
BHandHLYP	S_1_	3.04	0.54	H → L (0.95)
	S_2_	4.11	0.30	H → L+1 (0.91)
	S_3_	4.60	0.01	H-1 → L (0.82)
	S_4_	4.73	0.04	H-2 → L (0.41) H-3 → L (0.24)
PBE0	S_1_	2.64	0.35	H → L (0.98)
	S_2_	3.80	0.52	H → L+1 (0.95)
	S_3_	4.03	0.004	H-1 → L (0.60) H-2 → L (0.29)
	S_4_	4.08	0.003	H-2 → L (0.54) H-1 → L (0.34)
B3LYP	S_1_	2.51	0.31	H → L (0.98)
	S_2_	3.67	0.53	H → L+1 (0.91)
	S_3_	3.86	0.008	H-2 → L (0.48) H-1 → L(0.42)
	S_4_	3.92	0.01	H-1 → L (0.49) H-2 → L(0.38)
CAM-B3LYP	S_1_	3.07	0.52	H → L (0.92)
	S_2_	4.10	0.28	H → L+1 (0.87)
	S_3_	4.64	0.04	H-1 → L (0.56)
	S_4_	4.68	0.05	H-1 → L (0.23) H-4 → L (0.27)
ωB97XD	S_1_	3.09	0.54	H → L (0.90)
	S_2_	4.08	0.25	H → L+1 (0.82)
	S_3_	4.62	0.09	H-4 → L (0.48) H-2 → L (0.18)
	S_4_	4.76	0.03	H-1 → L (0.65)

**Table 2:** Frontier orbitals (HOMO-1 to LUMO+1) for monomer and dimer at the BHandHLYP/def2-SVP level.

Orbital	Monomer	Dimer
HOMO-1	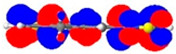	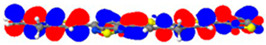
HOMO	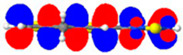	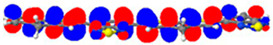
LUMO	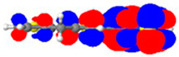	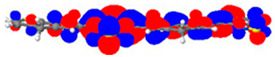
LUMO+1	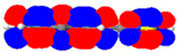	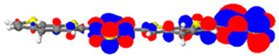

**Table 3:** The vertical excitation (VE) energies (eV), oscillator strengths (OS), and contributions of four lowest excited states for dimer in APC conformation at the BHandHLYP/def2-SVP, ωB97XD/6-31G*, and CAM-B3LYP/6-31G* levels. H and L indicate HOMO and LUMO, respectively.

Dimer	Excited States	VE	OS	Contribution
ADC(2)	S_1_	2.61	0.92	H → L (0.92)
	S_2_	3.22	0.16	H → L+1 (0.64) H-1 → L (0.18)
	S_3_	3.43	0.05	H-1 → L (0.69) H-1 → L+1 (0.12)
	S_4_	4.07	0.72	H → L+2 (0.72)
BHandHLYP	S_1_	2.35	1.64	H → L (0.92)
	S_2_	2.96	0.14	H → L+1 (0.73) H-1 → L (0.12)
	S_3_	3.34	0.013	H-1 → L (0.79)
	S_4_	3.80	0.61	H → L+2 (0.78) H-1 → L+3 (0.10)
CAM-B3LYP	S_1_	2.40	1.57	H → L (0.90)
	S_2_	3.02	0.15	H → L+1 (0.63) H-1 → L (0.17)
	S_3_	3.45	0.03	H-1 → L (0.70)
	S_4_	3.81	0.58	H → L+2 (0.74)
ωB97XD	S_1_	2.49	1.60	H → L (0.85)
	S_2_	3.13	0.17	H → L+1 (0.50) H-1 → L (0.22)
	S_3_	3.66	0.05	H-1 → L (0.50)
	S_4_	3.91	0.53	H → L+2 (0.65)

**Table 4:** The vertical excitation (VE) excitation energies (eV) and oscillator strengths (OSs) of the four lowest excited states for CPDTBT_N_ (N = 3–5) in APC. All the data were obtained at the BHandHLYP/def2-SVP, CAM-B3LYP/6-31G*, and ωB97XD/6-31G* levels of theory.

Oligomer	Excited State	BHandHLYP		CAM-B3LYP		ωB97XD	
		VE	OS	VE	OS	VE	OS
trimer	S_1_	1.87	2.68	1.96	2.58	2.06	2.61
	S_2_	2.27	0.26	2.33	0.02	2.41	0.025
	S_3_	2.65	0.20	2.73	0.22	2.85	0.24
	S_4_	2.79	0.016	3.00	0.015	3.29	0.07
tetramer	S_1_	1.77	3.86	1.86	3.73	1.97	3.77
	S_2_	2.10	0	2.16	0	2.25	0
	S_3_	2.32	0.17	2.38	0.17	2.48	0.17
	S_4_	2.64	0.10	2.73	0.21	2.85	0.23
pentamer	S_1_	1.71	5.02	1.81	4.86	1.92	4.91
	S_2_	1.97	0.004	2.05	0.004	2.14	0.004
	S_3_	2.20	0.30	2.26	0.28	2.36	0.28
	S_4_	2.35	0.04	2.41	0.04	2.50	0.04

**Table 5:** The energies (eV), oscillator strengths (OS), and contributions of four lowest excited states for hexamer in APC at the BHandHLYP/def2-SVP, ωB97XD/6-31G*, and CAM-B3LYP/6-31G* levels. H and L indicate HOMO and LUMO, respectively.

Method	Excited States	VE	OS	Contribution
BHandHLYP	S_1_	1.67	6.17	H → L (0.92)
	S_2_	1.88	0	H → L+1 (0.73) H-1 → L (0.12)
	S_3_	2.09	0.43	H-1 → L (0.79)
	S_4_	2.26	0	H → L+2 (0.78) H-1 → L+3 (0.10)
CAM-B3LYP	S_1_	1.77	5.98	H → L (0.54) H-1 → L+1 (0.19)
	S_2_	1.97	0	H → L+1 (0.34) H-1 → L (0.29)
	S_3_	2.16	0.41	H → L+2 (0.30)
	S_4_	2.32	0	H-1 → L+3 (0.29)
ωB97XD	S_1_	1.89	6.04	H → L (0.85)
	S_2_	2.07	0	H → L+1 (0.52) H-1 → L (0.22)
	S_3_	2.26	0.40	H-1 → L (0.50)
	S_4_	2.41	0	H → L+2 (0.65) H-1 → L+3 (0.16)

**Table 6:** Fitting parameters and extrapolated energy of S_1_ (E_1_) using the particle-in-a-box model and Kuhn’s equation at the BHandHLYP/def2-SVP, CAM-B3LYP/6-31G*, and ωB97XD/6-31G* Levels.

Equation	BHandHLYP			CAM-B3LYP			ωB97XD		
	A_1_	B_1_	E_1_ (eV)	A_1_	B_1_	E_1_ (eV)	A_1_	B_1_	E_1_ (eV)
Particle-in-a-box	1.29	2.03	1.29	1.41	1.88	1.41	1.54	1.80	1.54
Kuhn	2.91	−0.76	1.43	2.94	−0.73	1.53	3.01	−0.69	1.67

## Data Availability

Data are contained within this article.

## Supplementary Materials

The following supplementary materials can be downloaded at https://www.mdpi.com/article/10.3390/molecules29225348/s1, which includes computational detail, Figure S1: The transition density analysis of S_1_-S_4_ for dimer in the PC geometry at the ADC(2)/def2-SVP, BHandHLYP/def2-SVP, ωB97XD/6-31G*, CAM-B3LYP/6-31G* and B3LYP/def2-SVP levels. Figure S2: The transition density analysis of S_1_-S_4_ for hexamer in the PC geometry at the BHandHLYP/def2-SVP, ωB97XD/6-31G* and CAM-B3LYP/6-31G* levels. Table S1: The contributions of the double excitations for S_1_–S_4_ states of the monomer and the dimer were obtained at the ADC(2) level were listed in the following table. Table S2: Four orbitals (HOMO-1 to LUMO+1) for trimer. Table S3: Four orbitals (HOMO-1 to LUMO+1) for tetramer. Table S4: Four orbitals (HOMO-1 to LUMO+1) for pentamer. Table S5: HOMO-1, HOMO, LUMO and LUMO+1 for hexamer. Table S6: The energies (eV), oscillator strengths (OS) and contributions of four lowest excited states for dimer in the PC geometry at the ADC(2)/def2-SVP, BHandHLYP/def2-SVP, ωB97XD/6-31G*, B3LYP/def2-SVP and CAM-B3LYP/6-31G* levels. Table S7: The energies (eV) and oscillator strengths (OS) of four lowest excited states for trimer in the PC geometry at the BHandHLYP/def2-SVP, ωB97XD/6-31G* and CAM-B3LYP/6-31G* levels. Table S8: The energies (eV) and oscillator strengths (OS) of four lowest excited states for tetramer in the PC geometry at the BHandHLYP/def2-SVP, ωB97XD/6-31G* and CAM-B3LYP/6-31G* levels. Table S9: The energies (eV) and oscillator strengths (OS) of four lowest excited states for pentamer in the PC geometry at the BHandHLYP/def2-SVP, ωB97XD/6-31G* and CAM-B3LYP/6-31G* levels. Table S10: The energies (eV) and oscillator strengths (OS) of four lowest excited states for hexamer in the PC geometry at the BHandHLYP/def2-SVP, ωB97XD/6-31G* and CAM-B3LYP/6-31G* levels. Table S11: Transition densities of all the contributions for monomer. Table S12: Transition densities of all the contributions for dimer; M1 and M2 denotes two monomers: monomer1 and monomer2.
