# Supplementary material for: Investigating Excited States and Absorption Spectra of the Poly-cyclopenta-dithiophene-benzothiadiazole Oligomers (Poly-CPDTBT)—A Theoretical Study
Source: Molecules. 2024 Nov 14;29(22):5348. doi: 10.3390/molecules29225348 (PMC11596913; doi:10.3390/molecules29225348)
Supplement: Supplementary file 1 [file molecules-29-05348-s001.zip › molecules-3282385-supplementary.pdf]

Supplementary Materials for

Investigating Excited States and Absorption Spectra of the Poly-cyclopenta-dithiophene-benzothiadiazole Oligomers (Poly-CPDTBT)- A Theoretical Study

1. Computational detail

The excited states of the Poly-CPDTBT oligomers with two configurations were investigated by various theoretical methods. As discussed in many studies, the great caution should be taken to select the suitable methods to treat excited states reasonably. For example, the widely-used time-dependent density functional theory (TDDFT) often gives a good description on valence excitations, while certain functionals, especially the pure LDA and GGA functionals, strongly underestimate transition energies of charge-transfer excitations. The ratio of the HF exchange in hybrid functionals has a great influence on the excitation energies of CT states.[1-2] Therefore, the selection of the functionals is critical for the quality of the TDDFT treatments. In recent years, some studies suggested that the ADC(2) method is a useful method to give the reliable treatment of medium-sized systems with reasonable computational costs.[3] Particularly, as a propagation method, ADC(2) method does not suffer from the wrong treatment of the CT states. Thus, we took the ADC(2) method as our reference method to examine the performance of TDDFT with different functionals in the treatment of short oligomers.[1-2, 4]

We employed a two-step strategy to study the Poly-CPDTBT oligomers. In the first step, the extensive benchmark calculations with different methods were performed for monomer and dimer. Ground state geometric structures were optimized at both B3LYP and BHandHLYP levels of theory. For the excited state calculations, we

considered several functionals, including B3LYP (20% HF exchange), PBE0 (25% HF exchange) and BHandHLYP (50% HF exchange), in order to inspect the influence of Hartree-Fock (HF) exchanges. The basis set def2-SVP was employed in these calculations. The range-separated functional CAM-B3LYP and ωB97XD functional (with long-range correction and empirical dispersion) in combination with the 6-31G* basis set was also employed for comparison. All results were then carefully compared with values obtained at the ADC(2)/def2-SVP level of theory. The benchmark calculations (for monomer and dimer) show that the results obtained by the BHandHLYP, CAM-B3LYP and ωB97XD functionals are basically consistent with the ones at the ADC(2) level of theory (see below results). It should also be mentioned that the low-lying excited states mainly involve the CT excitation between two adjacent D-A units, instead of the long-distance CT transitions, see below discussions. In this sense, these three functionals reasonably capture the feature of such short-distance CT excitations between the two neighbor units (see below for details). Therefore, in the second step, these three functionals were used to treat the long oligomers. To reduce computational cost, the long oligomers ($CPDTBT_N$, N=3-6) were optimized at the BHandHLYP/SV level of theory. The excited states single-point TDDFT calculations of longer oligomers were performed at the BHandHLYP/def2-SVP, CAM-B3LYP/6-31G* and ωB97XD/6-31G* levels of theory.

In this work, the calculations at the TDDFT/CAM-B3LYP and TDDFT/ωB97XD levels were done with Gaussian 09 software[5] and all other calculations were performed within the TURBOMOLE 6.5 package.[6]

REFERENCES

1. Dreuw, A.; Head-Gordon, M., Single-Reference Ab Initio Methods for the Calculation of Excited States of Large Molecules. *Chem. Rev.* **2005**, *105*, 4009-4037.

2. Liu, W. L.; Settels, V.; Harbach, P. H. P.; Dreuw, A.; Fink, R. F.; Engels, B., Assessment of Td-Dft- and Td-Hf-Based Approaches for the Prediction of Exciton Coupling Parameters, Potential Energy Curves, and Electronic Characters of Electronically Excited Aggregates. *J. Comput. Chem.* **2011**, *32*, 1971-1981.

3. Plasser, F.; Aquino, A. J. A.; Hase, W. L.; Lischka, H., Uv Absorption Spectrum of Alternating DNA Duplexes. Analysis of Excitonic and Charge Transfer Interactions. *Journal of Physical Chemistry A* **2012**, *116*, 11151-11160.

4. Panda, A. N.; Plasser, F.; Aquino, A. J. A.; Burghardt, I.; Lischka, H., Electronically Excited States in Poly(P-Phenylenevinylene): Vertical Excitations and Torsional Potentials from High-Level Ab Initio Calculations. *Journal of Physical Chemistry A* **2013**, *117*, 2181-2189.

5. Frisch, M. J.; Trucks, G. W.; Schlegel, H. B.; Scuseria, G. E.; Robb, M. A.; Cheeseman, J. R.; Scalmani, G.; Barone, V.; Mennucci, B.; Petersson, G. A., et al. *Gaussian 09*, Gaussian, Inc.: Wallingford, CT, USA, 2009.

6. Ahlrichs, R.; Bar, M.; Haser, M.; Horn, H.; Kolmel, C., Electronic-Structure Calculations on Workstation Computers - the Program System Turbomole. *Chemical Physics Letters* **1989**, *162*, 165-169.

2. The examination of the symmetry

Results at the Cs ground-state geometry using the BHandHLYP and B3LYP methods indicate that all four lowest excited states (S_1-S_4) for $CPDTBT_N$ (N=1-6) belong to A' symmetry.

3. The contributions of the double excitation configurations

Table S1: The contributions of the double excitations for S_1-S_4 states of the monomer and the dimer were obtained at the ADC(2) level were listed in the following table.

	monomer	dimer
S_1	12%	12%
S_2	11%	13%
S_3	11%	15%
S_4	11%	13%

4. Four orbitals (HOMO-1 to LUMO+1) for trimer to pentamer

Table S2: Four orbitals (HOMO-1 to LUMO+1) for trimer

HOMO-1	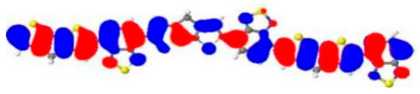
HOMO	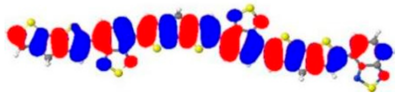
LUMO	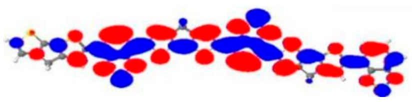
LUMO+1	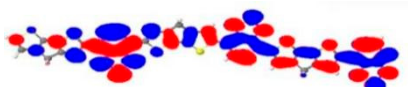

Table S3: Four orbitals (HOMO-1 to LUMO+1) for tetramer

HOMO-1	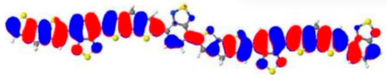
HOMO	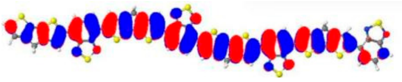
LUMO	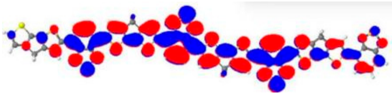
LUMO+1	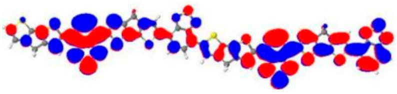

Table S4: Four orbitals (HOMO-1 to LUMO+1) for pentamer

HOMO-1	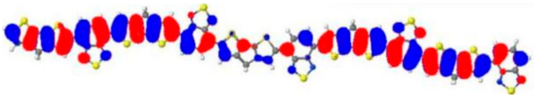
HOMO	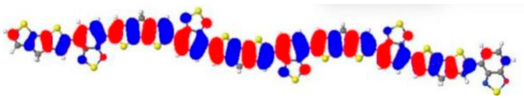
LUMO	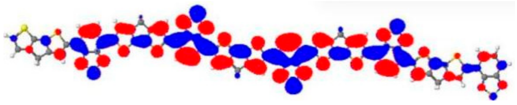
LUMO+1	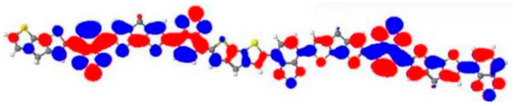

Table S5: HOMO-1, HOMO, LUMO and LUMO+1 for hexamer

HOMO-1	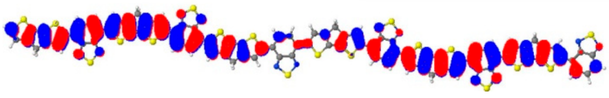
HOMO	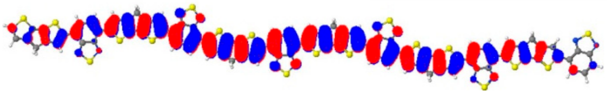
LUMO	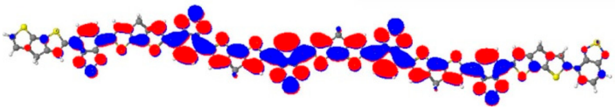
LUMO+1	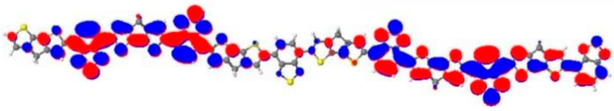

5. The energies (eV), oscillator strengths (OS) and contributions of four lowest excited states for oligomer in the PC configuration

Table S6: The energies (eV), oscillator strengths (OS) and contributions of four lowest excited states for dimer in the PC geometry at the ADC(2)/def2-SVP, BHandHLYP/def2-SVP, ωB97XD/6-31G*, B3LYP/def2-SVP and CAM-B3LYP/6-31G* levels

Method	Excited states	Energy	OS	Contribution
ADC(2)	S_1	2.64	1.69	H→L (0.91)
	S_2	3.24	0.20	H→L+1 (0.61)<br>H-1→L (0.21)
	S_3	3.47	0.04	H-1→L (0.64)<br>H-1→L +1(0.14)
	S_4	4.07	0.50	H→L+2 (0.71)
BHandHLYP	S_1	2.37	1.59	H→L (0.91)

	S_2	2.97	0.18	H→L+1 (0.71)<br>H-1→L (0.13)
	S_3	3.36	0.03	H-1→L (0.77)
	S_4	3.78	0.54	H→L+2 (0.76)
CAM-B3LYP	S_1	2.42	1.53	H→L (0.87)
	S_2	3.02	0.20	H→L+1 (0.61)<br>H-1→L (0.19)
	S_3	3.47	0.04	H-1→L (0.65)
	S_4	3.79	0.52	H→L+2 (0.72)
ωB97XD	S_1	2.46	1.49	H→L (0.85)
	S_2	3.06	0.23	H→L+1 (0.50)<br>H-1→L (0.22)
	S_3	3.61	0.06	H-1→L (0.48)<br>H-1→L+1(0.19)
	S_4	3.83	0.52	H→L+2(0.63)

Table S7: The energies (eV) and oscillator strengths (OS) of four lowest excited states for trimer in the PC geometry at the BHandHLYP/def2-SVP, ωB97XD/6-31G* and CAM-B3LYP/6-31G* levels

Excited state	BHandHLYP		CAM-B3LYP		ωB97XD	
	VE	OS	VE	OS	VE	OS
S_1	1.91	2.53	1.99	2.43	2.08	2.45
S_2	2.29	0.13	2.34	0.13	2.43	0.14
S_3	2.67	0.23	2.75	0.24	2.86	0.26
S_4	2.83	0.02	3.03	0.03	3.30	0.10

Table S8: The energies (eV) and oscillator strengths (OS) of four lowest excited states for tetramer in the PC geometry at the BHandHLYP/def2-SVP, ωB97XD/6-31G* and CAM-B3LYP/6-31G* levels

Excited state	BHandHLYP		CAM-B3LYP		ωB97XD	
	VE	OS	VE	OS	VE	OS
S_1	1.81	3.47	1.89	3.35	2.00	3.38
S_2	2.12	0.43	2.19	0.41	2.27	0.42
S_3	2.34	0.07	2.40	0.07	2.49	0.06
S_4	2.67	0.22	2.75	0.24	2.86	0.26

Table S9: The energies (eV) and oscillator strengths (OS) of four lowest excited states for pentamer in the PC geometry at the BHandHLYP/def2-SVP, ωB97XD/6-31G* and CAM-B3LYP/6-31G* levels

Excited state	BHandHLYP		CAM-B3LYP		ωB97XD	
	VE	OS	VE	OS	VE	OS
S_1	1.75	4.25	1.84	4.11	1.95	4.14
S_2	2.00	0.87	2.07	0.84	2.17	0.86
S_3	2.23	0.10	2.29	0.09	2.37	0.08
S_4	2.37	0.06	2.42	0.05	2.52	0.05

Table S10: The energies (eV) and oscillator strengths (OS) of four lowest excited states for hexamer in the PC geometry at the BHandHLYP/def2-SVP, ωB97XD/6-31G* and CAM-B3LYP/6-31G* levels

Excited state	BHandHLYP		CAM-B3LYP		ωB97XD	
	VE	OS	VE	OS	VE	OS
S_1	1.71	4.89	1.81	4.73	1.92	4.76
S_2	1.92	1.46	2.00	1.41	2.10	1.43
S_3	2.12	0.08	2.19	0.07	2.28	0.06
S_4	2.28	0.16	2.34	0.15	2.43	0.15

6. Transition densities of dimer and hexamer in the PC configurations

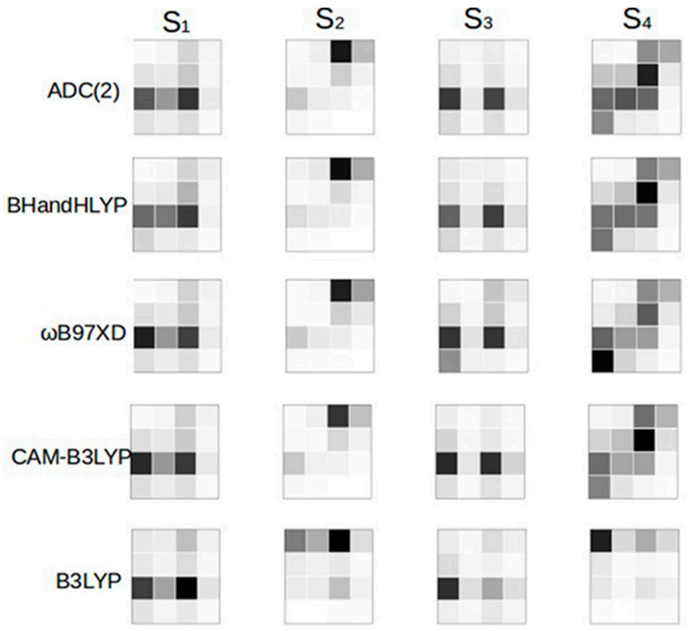

Figure S1: The transition density analysis of S_1-S_4 for dimer in the PC geometry at the ADC(2)/def2-SVP, BHandHLYP/def2-SVP, ωB97XD/6-31G*,CAM-B3LYP/6-31G* and B3LYP/def2-SVP levels

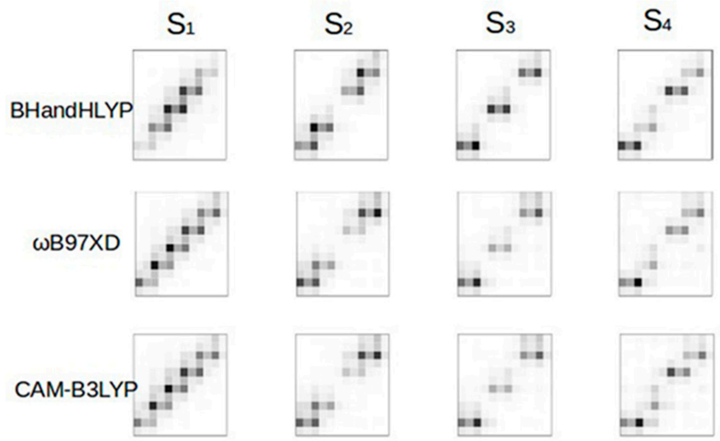

Figure S2: The transition density analysis of S_1-S_4 for hexamer in the PC geometry at the BHandHLYP/def2-SVP, ωB97XD/6-31G* and CAM-B3LYP/6-31G* levels

7. Transition densities of monomer and dimer

Table S11: Transition densities of all the contributions for monomer

		D-D	A-A	D-A	A-D
monomer	S_1	0.16	0.27	0.50	0.04
	S_2	0.53	0.15	0.18	0.10
	S_3	0.06	0.44	0.37	0.10
	S_4	0.13	0.42	0.36	0.05
	S_5	0.17	0.41	0.34	0.05
	S_6	0	0.93	0.03	0.02

Table S12: Transition densities of all the contributions for dimer; M1 and M2 denotes two monomers: monomer1 and monomer2

		M1-M1	M2-M2	M1-M2	M2-M1
dimer	S_1	0.42	0.20	0.07	0.28
	S_2	0.13	0.69	0.11	0.04
	S_3	0.38	0.10	0.14	0.37
	S_4	0.43	0.30	0.08	0.15
	S_5	0.24	0.29	0.38	0.04
	S_6	0.26	0.34	0.25	0.11
